# Does Social Support Matter in the Workplace? Social Support, Job Satisfaction, Bullying and Harassment in the Workplace during COVID-19

**DOI:** 10.3390/ijerph19084724

**Published:** 2022-04-13

**Authors:** Hjordis Sigursteinsdottir, Fjola Bjork Karlsdottir

**Affiliations:** School of Business and Science, University of Akureyri, 600 Akureyri, Iceland; fjolabjork@unak.is

**Keywords:** well-being at work, social support, harassment, job satisfaction, bullying

## Abstract

This study aims to examine social support at work amongst the employees of Icelandic municipalities and its relationship to job satisfaction, bullying and harassment. The study is based on an online survey conducted in 2021. A total of 4973 employees answered the questionnaire in part or in full after three reminders (57% response rate). The majority of the participants in the study were women (82%), but this gender ratio was representative of the population. The results show that social support gave an average score of 4.2 on a scale of 1–5; 87% of the participants were rather often or always satisfied with their job, 8% had experienced bullying at work, 2% had experienced sexual harassment and 3% had experienced gender-based harassment. Social support has a positive, moderately strong correlation with employee job satisfaction and a weak negative correlation with bullying at work. Based on the results, social support is an important factor related to the job satisfaction of employees and is a protective factor against bullying and sexual harassment at work. This finding demonstrates that managers and those responsible for employee well-being in the workplace should focus on social support at work, especially now that the psychosocial work environment is fragile because of COVID-19.

## 1. Introduction

Employees’ well-being in the workplace is important for both the employees and the company. Different variables can have negative impacts on employees’ health and well-being. Bullying and harassment are two factors that can have negative effects on employees’ health, well-being and job satisfaction [1], but research has shown that social support in the workplace can have a protective effect from negative factors in the working environment [2,3,4]. Social support at work includes helpful communication from the supervisor and colleagues and the provision of care, trust and respect [5,6]. The importance of viewing social support in the workplace in relation to workplace well-being is reflected in the third dimension of Karasek’s model in 1979 on job requirements and employment agency, i.e. workplace well-being is based on three dimensions: job requirements, agency and social support [6,7]. According to Rasmussen, Hansen, and Nielsen [8], if there is little social support from supervisors and co-workers, work can be stressful, but higher support contributes to well-being; they mention social support as one of the six aspects of the work environment that managers need to nurture in order to increase the well-being of workers in the workplace. In light of this, the main purpose of the present study is to examine the social support received by the employees of Icelandic local authorities and its association with job satisfaction, bullying and workplace harassment. The employees of Icelandic municipalities are an interesting group to explore, as the municipalities in Iceland as a whole are one of the largest employers in the country, with women comprising the vast majority of the staff [9].

Job satisfaction is a concept related to workplace well-being, which includes attitudes towards work and is both a subjective and emotional response of employees to their work [10]. Research shows that working conditions, such as social relationships with co-workers and supervisors, the possibility of promotion and opportunities for career development, have the greatest impact on job satisfaction and employee turnover amongst public servants [11,12,13,14]. Social support evidently has a positive impact on job satisfaction. Nevertheless, there is little research on the relationship between bullying and harassment and social support in the workplace in troubled times, such as now during the COVID-19 pandemic, and whether social support can be a protective factor against exposure to bullying and harassment.

## 2. Social Support and Job Satisfaction

There have been several definitions of social support in the workplace and its impact on employees. Cobb [15] defines social support as the information that an individual receives regarding how others care about this person and how this individual is part of a specific team and has the same commitments as the team. Hobfoll and Stokes [16] define social support as a relationship or social interaction that includes providing assistance and care to parties and forming a caring relationship between individuals or groups. Providing information is also part of social support and refers to counselling and guidance within the workplace to address increased demands and stress at work [17]. Social support increases employees’ expectations of the workplace and helps meet their needs for respect, emotional support and self-confidence, leading to higher job satisfaction. Previous research has shown that the higher the social support employees receive or perceive, the better they participate in their work and the more work commitment they show, leading to greater job satisfaction [18]. Rousseau and Aubé [19] point out that social support in the workplace can promote a positive work experience and thus foster greater commitment to one’s work. According to a study by Kiema-Junes et al. [20], social support in the workplace is linked to devotion to work, so the greater the support, the greater the devotion of an employee to their work.

Social support in the workplace can come from a supervisor or a co-worker. Support from co-workers refers to the extent to which co-workers provide social and emotional support and trust to other co-workers, as well as give assistance with other co-workers’ projects [6]. Support from supervisors can refer to emotional support, such as empathy, feedback and guidance, as well as support in terms of workplace resources and career progression [21]. McGuire [22] examines the effects of social support in the workplace and reports that the most common forms of support in the workplace are general conversations between co-workers, sharing and listening. Such support consists primarily of listening and providing employees an outlet for the release of difficult emotions, as social support in the workplace can contribute to employees’ improved well-being. This creates a good relationship between employees, making them improve their work environment and giving them the feeling that they belong to a team or a group in the workplace [23,24].

Studies show that women more often provide social support in the workplace than men do, but women also more often receive social support than men do [25,26]. Furthermore, research indicates that social support in the workplace has a more positive impact on men than on women [27,28,29]. The different effects of social support on women and men when providing and receiving social support have been attributed to women’s higher emotional stress, thus minimising the benefits of social support for women [29,30].

Job satisfaction is considered a key factor in the well-being of workers in the workplace; it affects employee absence, performance and turnover [10]. According to Locke [10], job satisfaction reflects a positive emotional state that is the result of the perceptions and experiences of employees regarding their work. Employees may feel varying degrees of satisfaction or dissatisfaction with the same job or with different parts of the job. Therefore, job satisfaction or dissatisfaction is a complex emotional response to the job. Kalleberg [31] states that work values combined with the benefits of various work aspects have a significant impact on job satisfaction. One of the sub-factors in his study, which he calls the third dimension, is workplace social support, which emerges through kindness and helpfulness. Saari and Judge [32] state that when a person thinks, they experience feelings related to what they think about. Previously, job satisfaction has often been measured using various influencing factors from the work environment, as Kalleberg [31] discusses, but Saari and Judge [32] emphasise that it is not necessary to ask many questions about several factors in the work environment to assess employee job satisfaction. It is sufficient to ask just one question, because such a measure would be appropriate and significant. Sometimes, it can be difficult to explain why employees who do the same job and are in the same work environment experience different levels of job satisfaction [31]. One of the factors examined by research is the effect of gender on job satisfaction, and the results differ. Some studies identify no gender differences in job satisfaction [33,34], whereas other studies identify higher job satisfaction in women than in men [35,36,37]. In the wake of the pandemic, for example, it is interesting to mention the results of Feng and Savani [38], who conducted a study on the job satisfaction of dual-career parents before and after the advent of the pandemic, during which workers were stuck at home (lockdown). The authors identified no difference in job satisfaction between women and men before the pandemic, but afterward, women were found to have lower job satisfaction than men did. 

The social support of supervisors and co-workers can have a major impact on job satisfaction [39,40,41]. A recent study by Pinna et al. [42] reports that social support from both supervisors and co-workers directly affects job satisfaction. A study by Kucharska and Bedford [40] also shows a strong link between social support and job satisfaction related to the willingness of employees to provide assistance and access to their knowledge to their colleagues; their work reports that job satisfaction creates a positive attitude and dedication to the workplace. Social support and job satisfaction have likewise been linked to quality of life. Yuh and Choi [43] examine the relationship between social support, job satisfaction and quality of life for preschool teachers. Their findings show a positive link between social support from both supervisors and co-workers and job satisfaction and that support from family members predicts quality-of-life once age and marital status are considered. They conclude that social support makes a significant difference in preschool teachers’ job satisfaction and overall quality of life.

A recent study on the effects of COVID-19 on the mental health of IT employees shows that remote working can affect both social interaction and job satisfaction and that there is a positive relationship between employee relations and job satisfaction, which is dependent on employees’ trust in their managers and co-workers. Therefore, maintaining and supporting social relationships are important [44]. Another study related to the COVID-19 pandemic and the changes in the work environment report that social support is one of the most important factors connected with employees’ well-being when working from home; more supportive colleagues make the participants feel less alone in the home office, and greater social support predicts better daily detachment from work [45]. These results indicate the importance of studying social support at work in the era of the COVID-19 pandemic.

## 3. Workplace Bullying and Harassment and Social Support

Scholars do not agree on how to define the concept of workplace bullying, but what most definitions have in common is that in workplace bullying, workers are exposed to repeated negative conduct or behaviour by other persons in the workplace [1]. The National Education Association [46] describes bullying as a systematic and chronic infliction of physical hurt and/or psychological distress upon another. Bullying involves a real or perceived power imbalance between the bully and the target. According to Icelandic regulation no. 1009/2015 [47] on measures against bullying, sexual harassment, gender-based harassment and violence in workplaces, bullying is reprehensible or repeated anticipatory conduct, act or behaviour that humiliates belittles, insults, injures, discriminates or inflicts on others and causes distress. Bullying is not linked to opinion disputes or conflicts of interest that can arise in the workplace. The same regulation states that sexual harassment is any form of sexual behaviour that is not appreciated or welcomed by the other person. The harassment has the purpose or effect of offending the dignity of the other person, particularly when the behaviour leads to threatening, hostile or humiliating situations, but the behaviour can be symbolic and/or physical. Gender-based harassment is offensive behaviour related to the gender of the person affected. The harassment can create situations that are threatening, hostile, humiliating or offensive to the person [47].

Icelandic research on bullying in the last decade demonstrates that prevalence of workplace bullying in Iceland has ranged from 10% to more than 20% [3,48,49]. In a recent study by Snorradottir et al. [48], which was conducted in Iceland, more than 20% of the participants reported being bullied at some point in their workplace, independent of the industries and the professions of these employees. Furthermore, the parties that were bullied in the workplace reported reduced job satisfaction and lower physical and mental health, and they were likelier to quit their jobs than those who had not experienced workplace bullying. The study showed that 16% of the participants had suffered sexual harassment, with 25% of women and less than 7% of men having experienced it, and 10% had experienced gender-based harassment, with more women having experienced it than men. Individuals who had been exposed to sexual harassment believed that their mental health was worse than that of those who had not been exposed to sexual harassment. A study conducted by Sigursteinsdottir [50] on bullying, harassment and violence in the workplace amongst members of the Teachers’ Union of Iceland reveals that more than 10% of the members had been exposed to workplace bullying between 2015 and 2017, with a much higher proportion for men than for women or over 12.4% of men and about 10% of women. In the same study, less than 2–3% had been exposed to sexual harassment or gender-based harassment, but the conclusion was similar for both genders.

According to previous research, victims of bullying believe that they receive less social support than other employees who have not been bullied, and they are less satisfied with their workplace supervisors [49,51]. A long-term study by Sigursteinsdottir et al. [3] amongst employees in the education sector and care services in Icelandic municipalities indicates that bullying and harassment increased because of the 2008 economic crisis. According to their research, prevalence of bullying was measured at 8% in 2010 but increased to 20% in 2015, sexual harassment ranged from 3.4% to 5.1% and gender-based harassment ranged from 3.7% to 6.7%. At the same time, the results show that employees at workplaces with greater job requirements, greater role disputes and less social support, where someone had been dismissed because of the economic crisis, were likelier to be bullied and harassed in the workplace than employees at other workplaces, which was the same for both genders. The findings show that the impact of cutbacks needs to be considered, especially now that economic hardship is rampant given the COVID-19 pandemic. As social support proved to be a protective factor against bullying and harassment, the authors believe that it is particularly important to emphasise social support in workplaces where one can expect the atmosphere to be dominated by stress and a lack of security because of COVID-19.

Ana [52] and Gransta [53] point out that one of the duties of employers is to ensure a working environment that is free of unwanted behaviour, such as bullying and harassment; most victims of workplace bullies state that the cause of bullying is largely or wholly based on the work environment [54]. The responsibility of employers is also stated in Icelandic regulation no. 1009/2015 [47], which indicates that the employer is obliged to prevent bullying, sexual harassment, gender-based harassment and violence in the workplace and to clarify to employees that such behaviours are prohibited. Kwan, Tuckey and Dollard [55] believe that workers can better deal with workplace bullying if the work environment and supervisors focus on the mental health of their employees, good workplace communication and the involvement of employees in issues that can affect their mental health and safety. Snorradottir and Tomasson [56] have recently investigated bullying in Icelandic workplaces, stating that cases received by the Labor Inspectorate had been going on for a while and that in most cases, the supervisor was identified as the bully. At the same time, the same study finds that if workplaces and supervisors fail to respond to bullying through targeted measures, bullying has significant consequences both for the employee, for example, increased sickness absence, and for the company, such as associated employee turnover and expenses.

Employees experiencing bullying or harassment in the workplace are both women and men, and the perpetrators are usually their co-workers or supervisors. Research has demonstrated that women working in male-dominated professions are likelier to be bullied and harassed in the workplace than other women are, and they are likelier to resign because of this. At the same time, the economic crisis has affected the rate of bullying and harassment in the workplace, and the proportion of those exposed to bullying or harassment in the workplace has been measured to be higher during periods of crisis [57,58]. Therefore, it is even more important to stay on top of the development of workplace well-being during difficult times, such as now with the COVID-19 pandemic.

Little research has focused on social support related to bullying and harassment in the workplace. Nielsen et al. [59] investigate whether workplace social support reduces the effects of bullying or protects workers from bullying, and they conclude that support from supervisors had a positive impact on both women and men, but support from co-workers had a more positive impact on women than on men. Furthermore, support outside the workplace did not seem to have any effect on protecting workers from bullying in the workplace. Their study included 10,627 employees, of which about 60% of the respondents were women and about 40% were men, in 96 different workplaces in Norway. The study provides evidence that social support reduces the impact of bullying on employees’ health and work ability, particularly in the case of supervisors’ support. Fang et al. [2] studied the impact of social support on bullying and the health of 238 nurses at a hospital in China and found that social support reduces the impact of bullying on workers’ health: the greater the social support in the workplace, the better the workers’ measured health. Rossiter and Sochos [4] examine the impact of social support on bullying and burnout amongst 222 employees in different companies and industries. Their study showed that the support of co-workers and supervisors reduces the impact of bullying and burnout at work and that social support includes both instrumental support and emotional support. It seems that instrumental support and emotional support reduce the effects of bullying, thus averting burnout. Instrumental support reduces the impact of workplace bullying by employee cynicism, whereas emotional support reduces the impact of bullying by averting emotional exhaustion.

**Figure 1:**
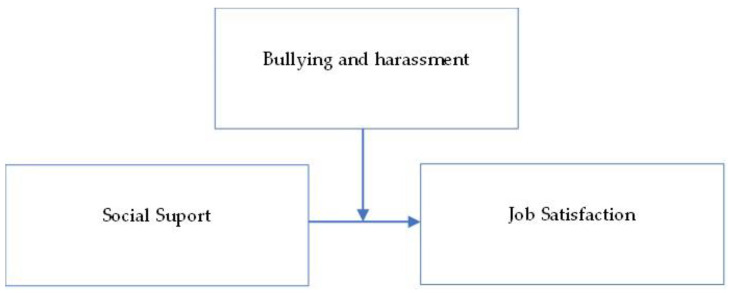
Conceptual moderation model for the relationships between the study variables.

Considering the above, it can be expected that unwanted workplace behaviour, such as bullying, sexual harassment and gender-based harassment, moderates an indirect association between social support and job satisfaction (see Figure 1). The overarching aim of this study was to determine the buffering role of social support in the relationship between perceived exposure to workplace bullying and harassment and job satisfaction. We pose the following research question: What is the relationship between social support at work and job satisfaction, and how does bullying and sexual and gender-based harassment affect that relationship in the current workplace? By answering this research question, we seek to obtain information about the buffering role of social support in the workplace. Furthermore, it was examined whether gender moderated the relationship between social support and job satisfaction, but gender was not a significant factor and was therefore excluded in the conceptual moderation model.

## 4. Materials and Methods

Data were collected using existing questionnaires sent electronically to the participants in this descriptive cross-sectional study. The Icelandic University Committee on Scientific Research approved the research.

### 4.1. Online Survey and Sample

An online survey was administered to the employees of 14 municipalities in Iceland. The human resource manager in each municipality provided the email addresses of all their employees with permanent positions (50% or higher). The questionnaire was sent to a total of 8790 employees, with a cover letter emphasising that the answers would not be traced back to individual participants and that full data confidentiality would be ensured. After three email reminders, 4973 employees answered the questionnaire partially (56.6%) and 4379 completely (49.8%), which is sufficient since the questionnaire was sent to all employees of the municipalities. The municipalities in Iceland are female-dominated workplaces where about 80% of the employees are women. The majority of the participants in this study were women (82.3%), which reflects the gender composition of the employees. The number of participants varied slightly in the analysis because of some missing values for the predictor variables.

### 4.2. Survey Questions

The survey designed for this study was based on two questionnaires: the Icelandic version of the General Nordic Questionnaire for Psychological and Social Factors at Work (QPS Nordic) [60] and the Health and Well-being of the Icelandic Nation questionnaire, published by the Icelandic Public Health Institute. In this study, we used four questions about support from supervisors and co-workers, one question about job satisfaction and three questions about bullying and harassment. In addition, one question was about gender (male, female, non-binary or other). Participants only marked male and female or skipped the question.

Support from superiors was measured using a two-item scale (‘If needed, can you get support and help with your work from your immediate superior/s?’ and ‘If needed, is your immediate superior/s willing to listen to your work-related problems?’) with five response options (1 = very seldom or never, to 5 = very often or always) (Cronbach’s alpha = 0.87). Support from co-workers was measured using the QPS Nordic two-item scale (‘If needed, can you get support and help with your work from your co-workers?’ and ‘If needed, are your co-workers willing to listen to your work-related problems?’) with five response options (1 = very seldom or never, to 5 = very often or always) (Cronbach’s alpha = 0.83). We also used these four questions as one variable describing social support at work (Cronbach’s alpha = 0.86).

Job satisfaction was measured using a one-item scale (‘On the whole, I am satisfied with my job’) with five response options (1 = totally disagree, to 5 = totally agree). Bullying and harassment were measured using the following three questions: (a) ‘Workplace bullying refers to hurtful and/or humiliating behaviour towards an individual. The behaviour is repeated and ongoing for some time—weeks, months or years. Have you been bullied in your current workplace in the past 12 months?’ (b) ‘Sexual harassment includes sexual activity, behaviour or comments directed at a person and against their consent or will. Have you experienced sexual harassment in your current workplace in the past 12 months?’ (c) ‘Gender-based harassment is hurtful or humiliating conduct towards women or men because of their gender, such as “women are…” and “men are…”, without being sexual. Have you suffered gender-based harassment in your current workplace in the past 12 months?’ These are yes/no questions.

### 4.3. Statistical Processing

The results are presented as numbers, percentages, averages and standard deviations. A Chi-square test was used to examine differences in support at work, job satisfaction and bullying and harassment by gender. Pearson’s R was used to evaluate the relationship between support at work and job satisfaction, and Spearman’s Rho was used to evaluate the relationship between support at work and bullying and harassment. The strength of the relationship was determined according to Cohen’s [61] guidelines, where *r* = 0.10 to 0.29 is small, *r* = 0.30 to 0.49 is medium and *r* = 0.50 to 1.0 is large. Analysis of the conceptual moderation model were conducted with the *PROCESS* tool v.4.0. In accordance with Aiken and West [62], the predictor variable (social support) was centred prior to the two-way interaction analysis. Statistical tests were conducted at a 5% level of significance, and data analyses were conducted using SPSS version 22.0 (IBM, Armonk, NY, USA).

## 5. Results

### 5.1. Social Support at Work

On a scale of 1–5, the average for social support at work was 4.2 (standard deviation (*Sd*) = 0.8). The average for support from superiors was 4.1 (*Sd* = 1.0) and that for support from co-workers was 4.2 (*Sd* = 0.8). Women (*M* = 4.2; *Sd* = 0.8) more often considered themselves to receive social support than men did (*M* = 4.0; *Sd* = 0.8), and this difference between genders was significant according to an independent *t*-test (*t*_(4422)_ = 5.2; *p* < 0.05). Women also considered themselves to receive more frequent social support from both superiors (*M* = 4.1/4.0; *Sd* = 1.0/1.0) (*t*_(4420)_ = 3.5; *p* < 0.05) and co-workers (*M* = 4.2/4.0; *Sd* = 0.8/0.9) (*t*_(4422)_ = 6.1; *p* < 0.05).

As shown in Table 1, 44.0% (±1.5%) of the participants very often or always received, if needed, support and help with work from their immediate superiors, and 28.6% (±1.3%) quite often received the same. Slightly more or 44.6% (±1.5%) very often or always received, if needed, support and help with work from co-workers, and 33.3% (±1.4%) quite often received the same. A higher proportion of women than men reported often receiving support from both superiors (*χ*^2^ _(4, N=4400)_ = 38.6; *p* < 0.05) and co-workers (*χ*^2^ _(4, N=4419)_ = 68.7; *p* < 0.05). About 48% (±1.5%) reported that, if needed, their immediate superiors were very often or always willing to listen to work-related problems, and nearly 32% (±1.4%) reported that their immediate superiors quite often showed the same willingness. Slightly fewer, or about 46% (±1.5%), reported that, if needed, co-workers were very often or always willing to listen to their work-related problems, and about 35% (±1.4%) reported that co-workers quite often showed the same willingness. A higher proportion of women than men reported that both immediate superiors (*χ*^2^ _(4, N=4407)_ = 10.8; *p* < 0.05) and co-workers were willing to listen to their work-related problems (*χ*^2^ _(4, N=4410)_ = 41,3; *p* < 0.05; *p* < 0.05).

### 5.2. Job Satisfaction, Bullying and Harassment at Work

Table 2 shows the results for job satisfaction by gender. Overall, the proportion of those who were very often or always satisfied with their job was 39.4 (±1.4%), and those who were rather often satisfied with their job was 48%. There was a significant difference in job satisfaction between women and men; in general, a higher proportion of women than men were more satisfied with their job (*χ*^2^ _(4, N=4482)_ = 11.3; *p* < 0.05).

Table 3 shows the results for bullying, sexual harassment and gender-based harassment in the current workplace in the past 12 months by gender. The results revealed that 7.9% (±0.8%) had been bullied in their current workplace in the past 12 months. A much lower proportion of employees had experienced sexual and gender-based harassment compared with bullying. The proportion of reported sexual harassment was 1.8 (±0.4%) and 3.2 (±0.5%) for gender-based harassment. No gender difference was found in exposure to bullying or sexual or gender-based harassment (*p* > 0.05), although the proportion for bullying was higher amongst men than amongst women, and the proportion for sexual and gender-based harassment was higher amongst women than amongst men.

### 5.3. Relationship between Social Support, Job Satisfaction, Bullying and Harassment in the Current Workplace

Table 4 shows the results of the correlation between social support, job satisfaction and bullying and harassment at work. There was a medium-strong positive correlation between social support and job satisfaction (*r* = 0.48) and a small negative correlation between social support and bullying (*r* = −0.22), indicating that the higher the social support, the higher the proportion of employees who are satisfied with their work. Furthermore, the results revealed a small negative correlation between job satisfaction and bullying (*r* = −0.18).

An independent *t*-test was conducted to examine the relationship between social support at work and bullying and harassment in the current workplace. The results showed that those who had been bullied in their current workplace in the past 12 months considered themselves less likely to receive social support (*M* = 3.4; *Sd* = 1.0) than those who had not been bullied (*M* = 4.2; *Sd* = 0.8), (*t*_(4386)_ = 17.8; *p* < 0.05). Those who had experienced sexual harassment in their current workplace also reported less social support at work (*M* = 3.9; *Sd* = 0.8) than those who had not been sexually harassed (*M* = 4.2; *Sd* = 0.8), (*t*_(4384)_ = 3.3; *p* < 0.05). Furthermore, social support was measured to be lower amongst those who had experienced gender-based harassment (*M* = 3.9; *Sd* = 1.1) than amongst those who had not experienced gender-based harassment (*M* = 4.2; *Sd* = 0.8), (*t*_(4379)_ = 4.3; *p* < 0.05).

Table 5 reports the results of the conceptual moderation models. In model 1, social support and bullying and the interaction term explained a total of 25% of the variance in job satisfaction. The overall model was significant (*F*_(3, 4368)_ = 478.52; *p* < 0.01). All three predictors, social support (*b* = 0.41, *t*_(4368)_ = 29.41, *p* = 0.001), bullying (*b* = −0.21, *t*_(4368)_ = −4.49, *p* = 0.001) and social support x bullying (*b* = 0.20, *t*_(4368)_ = 4.97, *p* < 0.001), yielded significant contributions to the variance in job satisfaction. The findings showed that exposure to bullying interacts with social support and job satisfaction. Follow-up analyses of simple slopes revealed that social support was strongly related to job satisfaction among employees who had experienced bullying at work (*b* = 0.61, *t*_(4368)_ = 16.35, *p* < 0.001) and moderately strongly related to employees who had not experienced bullying at work (*b* = 0.41, *t*_(4368)_ = 29.41, *p* < 0.001).

In model 2, social support, sexual harassment and the interaction term explained a total of 24% of the variance in job satisfaction. The overall model was significant (*F*_(3, 4368)_ = 448.3; *p* < 0.001). All three predictors, social support (*b* = 0.48, *t*_(4368)_ = 36.56, *p* = 0.001), sexual harassment (*b* = −0.16, *t*_(4368)_ = −1.96, *p* = 0.05) and social support × sexual harassment (*b* = −0.36, *t*_(4368)_ = 4.61, *p* < 0.001), yielded significant contributions to the variance in job satisfaction. The findings showed that exposure to sexual harassment interacts with social support and job satisfaction. Follow-up analyses of simple slopes revealed that social support was moderately strongly related to job satisfaction among employees who had not experienced sexual harassment at work (*b* = 0.48, *t*_(4368)_ = 36.56, *p* < 0.001) but not to those who had experienced sexual harassment (*b* = 0.12, *t*_(4368)_ = 1.51, *p* = 0.132).

In model 3, social support, gender-based harassment and the interaction term explained a total of 23% of the variance in job satisfaction. The overall model was significant (*F*_(3, 4361)_ = 439.95; *p* < 0.001). Only social support (*b* = 0.46, *t_(_*_4361)_ = 34.52, *p* = 0.001), and gender-based harassment (*b* = −0.12, *t*_(4361)_ = −1.00, *p* = 0.05) yielded significant contributions to the variance in job satisfaction and social support × gender-based harassment (*b* = 0.11, *t*_(4361)_ = 1.90, *p* = 0.057) did not.

## 6. Discussion and Conclusions

The aim of this study was to examine social support in the workplace amongst Icelandic municipal employees and its association with job satisfaction, bullying and sexual and gender-based harassment in the workplace. The results show that social support has a positive, moderately strong relationship with employee job satisfaction and a weak negative correlation with bullying in the current workplace. These findings support those of other studies that found that social support is associated with high job satisfaction [39,40,42,43] and that it is a protective factor against unwanted workplace behaviour, such as bullying and sexual harassment. These results are also consistent with those of Sigursteinsdottir et al. [3] and Nielsen et al. [59]. Workers who have suffered bullying and/or harassment in the workplace believe that they receive less frequent social support in the workplace from superiors and co-workers compared with those employees who have not been exposed to such behaviours [48,49].

Over the past decade, managers have become increasingly aware of how important it is to promote social support in the workplace, which can lead to higher job satisfaction and better communication between employees. It is important that the work environment is characterised by mutual respect in all communication and that employees are protected from bullying and harassment in the workplace, as Ana [52] and Gransta [53] emphasise. Kwan et al. [55] cast the responsibility more on superiors and underline that it is their role to create a workplace in which social support and job satisfaction are nurtured and to provide protection against bullying and harassment. This is in accordance with the Regulation on Actions against Bullying, Sexual Harassment, Gender-Based Harassment and Workplace Violence [47] in Iceland, which states that it is not permitted to bully or harass employees and that it is the responsibility of superiors to act on such issues and ensure the well-being of employees in the workplace.

The results show that the participants of the study believed that their superiors and co-workers provided them, in most cases, with support and help with tasks, if needed, and they listened to the problems that employees had at the workplace. These are positive outcomes for Icelandic municipal managers because, as Lan et al. [18] point out, employees experience of workplace social support leads to positive feelings towards their work. Moreover, Kiema-Junes et al. [20] state that social support is associated with dedication to work, meaning that when the social support is high, the devotion to work is also high. The results further show that a higher proportion of women than men felt that they received social support more often at work from both superiors and co-workers. This finding is supported by the 2013 study of Sigursteinsdottir [49], which reported that women evaluated that they receive more social support than men do. The works of Beerh et al. [25] and Fuhrer et al. [26] support these findings, with the addition that men get more out of the social support they receive than women do, which is interesting but Schwarzer et al. [29] and Walen and Lachman [30] explain different effects of social support by higher emotional stress of women when providing and receiving social support from co-workers, thus minimising the benefits of social support for women.

In this study, the incidence of bullying was about 8%; sexual harassment, 2%; and gender-based harassment, 3%. The proportion of people who reported being bullied in the Icelandic municipal workplace is therefore much lower than that reported by other Icelandic studies in the last few years, in which the rate ranged from 10% to 20% [3,48,49,50]. In terms of sexual harassment and gender-based harassment, these findings are similar to those of Sigursteinsdottir’s (2017) study [50] amongst members of the Teachers’ Union of Iceland. It is not surprising because a large proportion of Icelandic municipal employees are primary school and preschool teachers. The results also showed no significant differences in bullying or harassment by gender. However, a study conducted by Snorradóttir et al. [48] amongst people from various professions showed that 16% of the participants have been exposed to sexual harassment in the workplace, 10% to gender-based harassment and about 20% to bullying. It is therefore likely that amongst large professions working in municipal work contexts, such as primary school and preschool teachers, the prevalence of bullying and harassment is less than that amongst other professions. Bullying and harassment by students in primary school and pre-school is often a problem that teachers must deal with on a day-to-day basis, and they might therefore be more aware of bullying and harassment and have a better understanding of it and capability to address and deal with it at the workplace. The findings could also mean that there is less tolerance among employees regarding bullying and harassment; thus, the organisational culture could explain the lower prevalence of bullying and harassment in our study.

The relevance of the study is that it is a population study in which all employees of 14 municipalities in Iceland were invited to participate. We received responses from more than half of the employees (57%), which is good, and the study participants also reflected the gender imbalance of municipal workplaces. Certain limitations should though be kept in mind when interpreting the results. First, the results reflect the perception of municipal workers where most employees are women and applying the results to other workplaces in the private sector where the gender ratio is close to equal, or employees are predominately male, should be done with caution. A replication of the current study using workplaces with a different gender ratio is needed to validate generalisability of the current findings. Second, as the study is cross-sectional, it shows results at one point in time, during the COVID-19 pandemic. Exploring further whether and how the attitudes of the employees evolve during course of the pandemic, which presumably affected the workplaces of the municipalities, would give valuable insight into the relationship between social support at work and job satisfaction. There is further opportunity to repeat and compare the study when the pandemic is over. Third, the study took use of self-reported measures, reflecting respondents’ perception and not objective information. To date, objective measures of structural social support have not been developed but would be an interesting addition to current methods, especially given the opportunity to compare perceived and objective support and its impact on job satisfaction. Further, as big part of municipality employees is working in education and care, the use of self-report measures imposes a possible bias of socially positive responses. Fourth, as it was not in the scope of the current manuscript, organisational characteristics that could influence the relationship between workplace support and job satisfaction were not included in the analysis. It would be valuable for future research to include variables such as role conflict, leadership values or organisational culture.

The practical value of the research is the knowledge it contributes regarding the importance of workplace social support and its association with unwanted behaviours, such as bullying and workplace harassment. It can be concluded from the research results that the social support of co-workers and supervisors is an important factor related to the job satisfaction of municipal employees and is a protective factor in the prevention of bullying and harassment in the workplace. This tells us that supervisors and those responsible for the well-being of employees in the workplace should focus on workplace social support, especially during difficult times, such as now with the COVID-19 pandemic, and in workplaces where the psychosocial work environment is difficult. It is of importance to design policies that consider the protective factor of social support and the relationship of social support with job satisfaction.

## Figures and Tables

**Table 1:** Social support at work by gender.

		Participants	Very Seldom or Never %	Rather Seldom %	Sometimes %	Rather Often %	Very Often or Always %
Support and help from superior/s	Total	4401	2.8	6.2	18.4	28.6	44.0
	Female	3601	2.9	5.5	17.6	28.2	45.8
	Male	799	2.4	9.3	21.8	30.8	35.8
Support and help from co-workers	Total	4420	1.5	3.5	17.0	33.3	44.6
	Female	3619	1.6	2.7	15.9	33.5	46.3
	Male	800	0.9	7.2	22.1	32.5	37.3
Superior/s listen to work-related problems	Total	4408	2.7	4.5	13.0	31.7	48.1
	Female	3607	2.6	4.5	12.8	30.9	49.2
	Male	800	2.9	4.3	14.0	35.4	43.5
Co-workers listen to work-related problems	Total	4411	1.4	3.3	13,8	35.3	46.3
	Female	3611	1.4	2.6	13.5	34.8	47.8
	Male	799	1.5	6.4	15.0	37.5	39.5

**Table 2:** Job satisfaction by gender.

		Participants	Very Seldom or Never %	Rather Seldom %	Sometimes %	Rather Often %	Very Often or Always %
Job satisfaction	Total	4483	1.1	2.7	8.9	48.0	39.4
	Female	3677	0.9	2.8	8.9	47.5	39.9
	Male	805	2.0	2.1	8.3	50.3	37.3

**Table 3:** Bullying and harassment in the current workplace past 12 months by gender.

		Participants	Yes %	No %
Bullying at work	Total	4393	7.9	92.1
	Female	3592	7.6	92.4
	Male	800	9.5	90.5
Sexual harassment at work	Total	4391	1.8	98.2
	Female	3590	1.9	98.1
	Male	800	1.3	98.8
Gender-based harassment at work	Total	4386	3.2	96.8
	Female	3585	3.3	96.7
	Male	800	2.9	97.1

**Table 4:** Correlation between social support, job satisfaction, bullying, sexual harassment and gender-based harassment.

	1	2	3	4	5	6	7
1. Social support	-	-	-	-	-	-	-
2. Support from superiors	-	-	-	-	-	-	-
3. Support from co-workers	-	-	-	-	-	-	-
4. Job satisfaction	0.480 **	0.472 **	0.394 **	-	-	-	-
5. Bullying	−0.218 **	−0.216 **	−0.172 **	−0.182 **	-	-	-
6. Sexual harassment	−0.038 *	−0.047 **	−0.023	−0.022	0.093 **	-	-
7. Gender-based harassment	−0.045 **	−0.040 **	−0.028	−0.026	0.161 **	0.305 **	-

** Correlation is significant at the 0.01 level; * Correlation is significant at the 0.05 level.

**Table 5:** Linear models of predictors of job satisfaction.

Model	Variable	*β*	SE *β*	*t*	*R* ^2^
1					0.2475
	Constant	4.25 (4.23, 4.27)	0.01	387.43 **	
	Social Support	0.42 (0.39, 0.44)	0.01	29.41 **	
	Bullying	−0.21 (−0.30, −0.12)	0.05	−4.49 **	
	Social Support * Bullying	0.20 (0.12, 0.28)	0.04	4.97 **	
2					0.2355
	Constant	4.23 (4.20, 4.25)	0.01	395.82 **	
	Social Support	0.48 (0.45, 0.50)	0.01	36.56 **	
	Sexual harassment	−0.16 (−0.33, −0.00)	0.08	−1.96 *	
	Social Support * Sexual harassment	−0.36 (−0.51, −0.21)	0.08	−4.61 **	
3					0.2325
	Constant	4.23 (4.21, 4.25)	0.01	392.42 **	
	Social Support	0.46 (0.43, 0.49)	0.01	34.52 **	
	Gender-based harassment	−0.12 (−0.25, −0.00)	0.06	−1.99 *	
	Social Support * Gender-based harassment	−0.11 (−0.00, 0.22)	0.06	1.90	

** *p* < 0.01; * *p* < 0.05.

## Data Availability

Not applicable.
